# Identification of Synovial Fibroblast-Associated Neuropeptide Genes and m6A Factors in Rheumatoid Arthritis Using Single-Cell Analysis and Machine Learning

**DOI:** 10.1155/2022/5114697

**Published:** 2022-02-09

**Authors:** Jianwei Xiao, Xu Cai, Rongsheng Wang, Weijian Zhou, Zhizhong Ye

**Affiliations:** ^1^Shenzhen Futian Hospital for Rheumatic Diseases, China; ^2^Department of Rheumatology, Shanghai Guanghua Hospital of Integrated Traditional and Western Medicine, China; ^3^Rheumatism Dept. Yunnan Provincial Hospital of Traditional Chinese Medicine, China

## Abstract

**Objectives:**

Synovial fibroblasts (SFs) play an important role in the development and progression of rheumatoid arthritis (RA). However, the pathogenic mechanism of SFs remains unclear. The objective of this study was to investigate how neuropeptides and N6-methyladenosine (m6A) played an important role in the underlying pathogenic processes of SFs that contribute to the development of RA.

**Methods:**

Single-cell RNA sequencing data were examined using single-cell analysis and machine learning. SF subgroups were identified based on the clustering and annotation results of the single-cell analysis. Moreover, cell–cell communication was used to analyse neuropeptide-related receptor and ligand pairs on the surface of SF cell membranes. Machine learning was used to explore the m6A factors acting on these neuropeptide genes.

**Results:**

NPR3, GHR, BDKRB2, and CALCRL, four neuropeptide genes, were shown to be differently expressed among SF subgroups. Further investigation of receptor–ligand interactions found that NPR3 (in conjunction with NPPC, OSTN, NPPB, and NPPA) and GHR (in conjunction with GH1 and GH2) may have a role in SF interactions. As predicted by machine learning, *IGFBP2* and *METTL3* were identified as key factors regulating m6A of *NPR3* and *GHR*. The expression levels and enrichment pathways of *METTL3* and *IGFBP2* were different among SF subgroups.

**Conclusions:**

Single-cell analysis and machine learning efficiently identified neuropeptide genes and m6A factors that perform important regulatory functions in RA. Our strategy may provide a basis for future studies to identify pathogenic cell subpopulations and molecular mechanisms in RA and other diseases.

## 1. Introduction

Rheumatoid arthritis (RA) is a chronic immune disease that primarily affects the synovial lining of joints and affects roughly 1% of the world's population [1, 2]. RA can present with a variety of clinical symptoms and modes of progression. The etiology of RA is unclear. It is usually thought to occur in response to a combination of genetic susceptibility and environmental factors. The typical pathology of RA is the proliferation of synovial fibroblasts (SF), also termed fibroblast-like synoviocytes (FLS), in the joints and the formation of granulations that erode and destroy the articular cartilage [3]. SF maintains the persistence of inflammation and is driven by multiple epimodification modalities [4]. In addition, fibroblasts can switch from early immunosuppressive to stimulatory in response to relevant factors to promote RA development [5]. Therefore, SF may serve as a potential and safe therapeutic target [6].

Several previous studies have explored the relationship between neuropeptides and RA. High NPY levels are an independent marker of RA activity [7]. Patients with RA have increased levels of SP in synovial fluid and serum, and NK-1R was adjusted upward in SF [8]. Symptoms of the disease can be reduced after sympathetic tone decreases in RA patients [9]. In addition, both excessive and insufficient production of neuropeptides may be harmful to joint cartilage [10]. The treatment carried out for neuropeptides also showed good results in arthritis experiments in mice [11]. Therefore, neuropeptides may play an important role in the development of RA.

Epistatic modifications can stably alter gene expression without changing the nucleic acid base ordering. DNA methylation and histone modifications all play important roles in RA [12]. N6-Methyladenosine (m6A) is the most abundant form of mRNA modification, and it plays an important regulatory role in cancer as well as immune diseases in the form of posttranscriptional modifications [13–15]. RNA methylation involves a variety of m6A readers, writers, and erasers [16, 17]. These enzymes or factors are involved in almost every process of mRNA metabolism, as well as playing a role in various physiological processes [18]. RNA m6A is similarly involved in several immune functions, including immune recognition and activation, as well as cell fate determination [19]. The relationship between RA, as a systemic autoimmune disease, and m6A remains unclear [20].

In summary, both neuropeptides and m6A may play an important role in the development of RA. However, no research has been done at the interaction between neuropeptides and m6A in RA. The development of single-cell analytic methods has resulted in more efficient research of SF at the cellular level in recent years [21]. Moreover, bioinformatics approaches such as cellular communication enable the prediction of cell-cell interaction relationships [22]. As a result, we attempted to examine the relationship between neuropeptides and m6A in RA through single-cell analysis and machine learning techniques.

## 2. Materials and Methods

### 2.1. Data Acquisition and Preprocessing

By searching “rheumatoid arthritis (RA)” in the GEO database, we obtained two datasets (including GSE109449 and GSE21959). GSE109449 contains synovial fibroblast transcript data from two RA and two OA (osteoarthritis) patients [23]. The single-cell RNA-seq data obtained were used to analyse the relationships between subpopulations of fibroblasts. The GSE21959 dataset provided bulk RNA-seq data from six RA and six adult donors' synovial tissues. Based on the GSE21959 dataset, differentially expressed genes between RA and HC were screened [24]. The data from single-cell RNA-seq was filtered to ensure that genes were expressed in at least three cells and that each cell had at least 200 genes expressed. Afterwards, quality control of the RNA data was assessed and compared across samples. RNA data was normalised by log transformation and feature scaling. “edgeR” was then applied to the bulk RNA count for differentially expressed gene analysis [25]. In addition, we performed real-time RT-PCR (qRT-PCR) validation in a clinical cohort at our hospital. Informed consent was obtained from all our patients. The study was in line with the principles set out in the “Helsinki Manifesto.”

### 2.2. Neuropeptide-Related Genes and m6A Factors

Neuropeptide-related genes included in this study included both neuropeptides and neuropeptide receptors. Through a literature review, 53 neuropeptide-related genes were included in this study (*NPY*, *NPY1R*, *NPY2R*, *NPY4R*, *NPY5R*, *CALCA*, *CALCB*, *CALCR*, *CALCRL*, *CRCP*, *ADCYAP1*, *ADCYAP1R1*, *PACAP*, *GAL*, *GALR1*, *GALR2*, *VIP*, *VIPR1*, *VIPR2*, *NPPA*, *NPPA-AS1*, *NPR1*, *NPR3*, *NPR2*, *NPPB*, *NPPC*, *PDYN*, *PDYN-AS1*, *AGT*, *AGTR1*, *AGTR2*, *SP*, *SN*, *ENK*, *EP*, *DYN*, *SIGMAR1*, *OPRK1*, *OPRD1*, *OPRM1*, *OPRL1*, *TACR1*, *BDKRB2*, *BDKRB1*, *NPSR1*, *NTS*, *NTSR1*, *NTSR2*, *GH*, *GHR*, *PRL*, *PRLR*, and *TAFA4*). In addition, 23 m6A factors, including 8 writers, 13 readers, and two erasers, were also incorporated.

### 2.3. Single-Cell Data Analysis

For single-cell analysis, we utilized the Seurat program (version 4.0.4), which included data cleaning, dimensionality reduction, clustering, and cell annotation, similar in previous research [26–28]. After quality control and normalisation, we first identified highly variable genes in all cells. Principal component analysis (PCA) was then performed for feature extraction based on highly variable genes. The “ElbowPlot” function was used to filter the number of principal components (PCs). Finally, based on the selection of PCs, single cells were clustered and visualised in a Uniform Manifold Approximation and Projection (UMAP) fashion. The marker genes for each cluster were calculated and filtered by the “FindAllMarkers” function. The cell clusters were annotated according to the surface protein markers screened via flow cytometry in the original literature. Moreover, based on the UMAP algorithm, we projected the original tissue information of cells into clusters.

### 2.4. Ligand Receptor Analysis

We used CellPhoneDB to screen for possible cell–cell communication neuropeptide-related molecules in SFs as previous research [29–31]. The gene expression matrix of single-cell RNA sequencing (scRNA-seq) and the annotation of the ligand–receptor relationship pairs from the literature allowed systematic access to intercellular communication networks. Neuropeptide-related genes identified via single-cell analysis were used to filter these receptor and ligand molecules using intersectional analysis.

### 2.5. Machine Learning-Based Variable Screening

Based on the cellular communication analysis, we obtained significant neuropeptide-related genes. Furthermore, m6A regulators that may act on neuropeptide-related genes were predicted by using the random forest model (RFM) and the support vector machine–recursive feature elimination (SVM-RFE). As indicated in prior investigations, RFM and SVM-RFE were used [32–34]. The m6A genes in the model were ranked by significance using the random forest model. SVM-RFE was used for screening m6A genes most closely related to neuropeptide genes [35].

### 2.6. Key m6A Regulatory Genes Were Identified by Intersection Analysis

Differential analysis of bulk RNA-seq between normal and RA databases screened out differentially expressed genes (DEGs) in RA. Subsequently, DE m6A-regulated genes in bulk RNA were identified. The m6A regulatory genes that are both differentially expressed in RA samples and associated with neuropeptides were identified as key m6A regulatory genes.

### 2.7. Gene Set Enrichment Analysis (GSEA)

GSEA was used to analyse differentially active functional pathways in cells with differential expression of m6A-regulated genes. A random sample swap (*n* = 1000) was performed to calculate the *P* value. We used the clusterProfiler package to perform GSEA [36].

### 2.8. Quantitative Reverse Transcription Polymerase Chain Reaction (qRT-PCR)

Total RNA was extracted using TRIzol reagent (Invitrogen, MA, USA), and qRT-PCR was preformed using SYBR Premix ExTaq (Takara Bio Inc., Japan). After obtaining the comparative cycle threshold (Ct), the *ΔΔ*Ct method was used for normalisation of mRNA expression [37]. Glyceraldehyde 3-phosphate dehydrogenase expression was used as an internal reference primer.

### 2.9. Statistical Analysis

All tests were two-sided, and *P* < 0.05 was considered significant. All analyses were performed using the R software (version 4.0.2, R Foundation for Statistical Computing, Vienna, Austria). The receiver operating characteristic (ROC) curves were created using the “pROC” package to evaluate the diagnostic performance of candidate m6A regulatory genes.

## 3. Results

### 3.1. ScRNA-seq Reveals a Heterogeneous Phenotype of SFs

**Figure 1:**
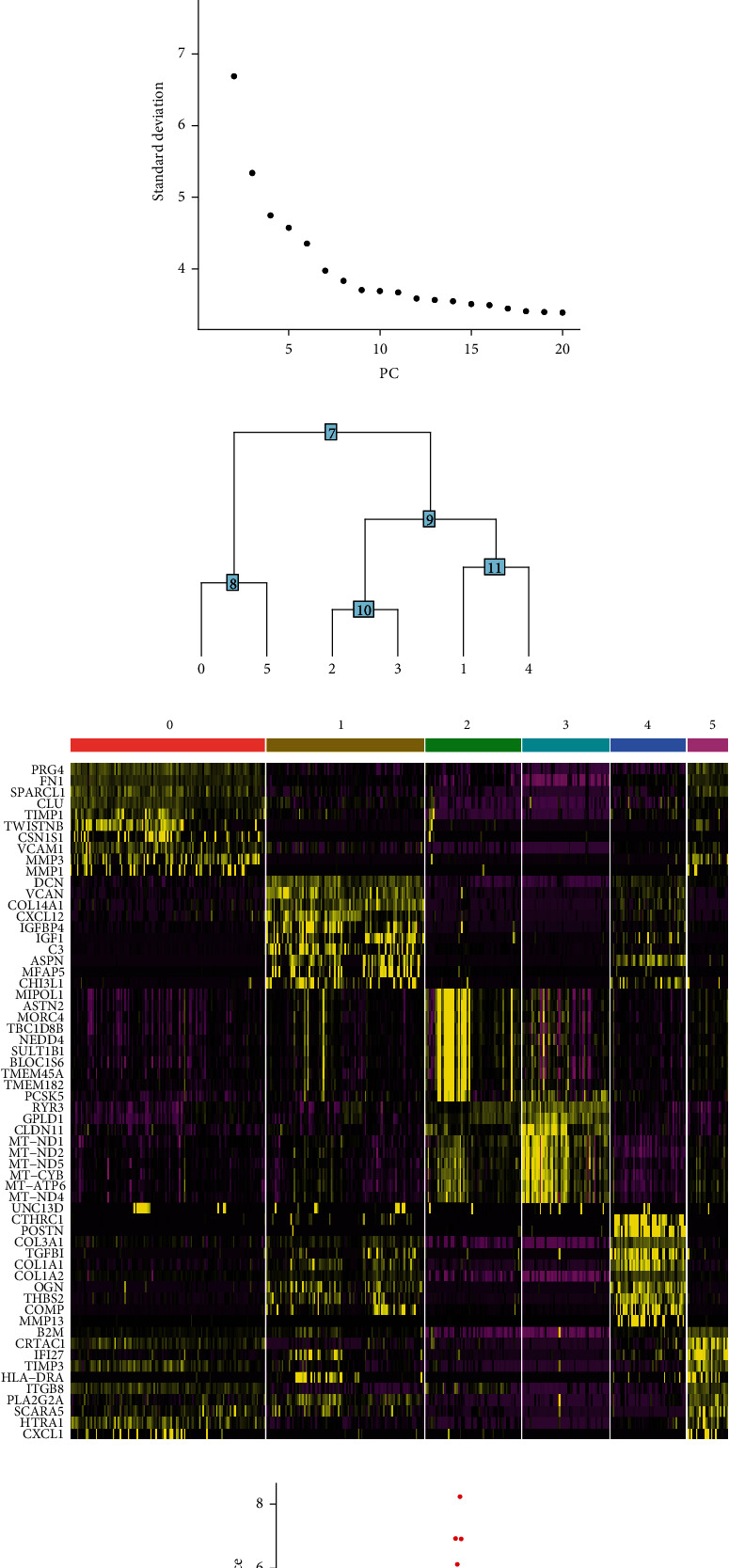
Single-cell analysis preprocessing and downscaling and clustering process for GSE109449. (a) Data accusations after filtering of genes and cells. (b) Elbow plot showing that the inflection point occurs, suggesting that the first nine PCs can contain most signals. (c) All included cells are grouped into six clusters based on a clustering tree constructed based on the selected nine PCs. (d) Heat map showing the expression of the top 10 marker genes of the six clusters in single-cell samples. (e) Volcano plot showing differences in the expression of neuropeptide-related mRNAs among clustering-related marker genes. Abbreviation: PC: principal component.

To examine the possible role of SFs in RA, four samples from the GSE109449 dataset were used to explore the heterogeneity of fibroblasts.  Figure 1(a) shows the quality control results of these cells as described in Seurat's manual [38]. A total of 384 cells were screened for inclusion in the subsequent analysis. Analysis of variance was performed to compare the genetic variability of SFs. Among the neuropeptide-related genes, significant transcriptional differences were found in *NPR3*, *BDKRB2*, *GHR*, and *CALCRL* (Figure 1(e)). The elbow plot demonstrated that the best clustering results of applying the first nine PCs can contain most signals (Figure 1(b)). The results of the clustering tree are shown in Figure 1(c), which shows a total of six cell clusters. The heat map revealed the top 10 most important cell marker genes in each of these six cell clusters (Figure 1(d)).

### 3.2. Fibroblast Subpopulations and Neuropeptide-Related Genes

**Figure 2:**
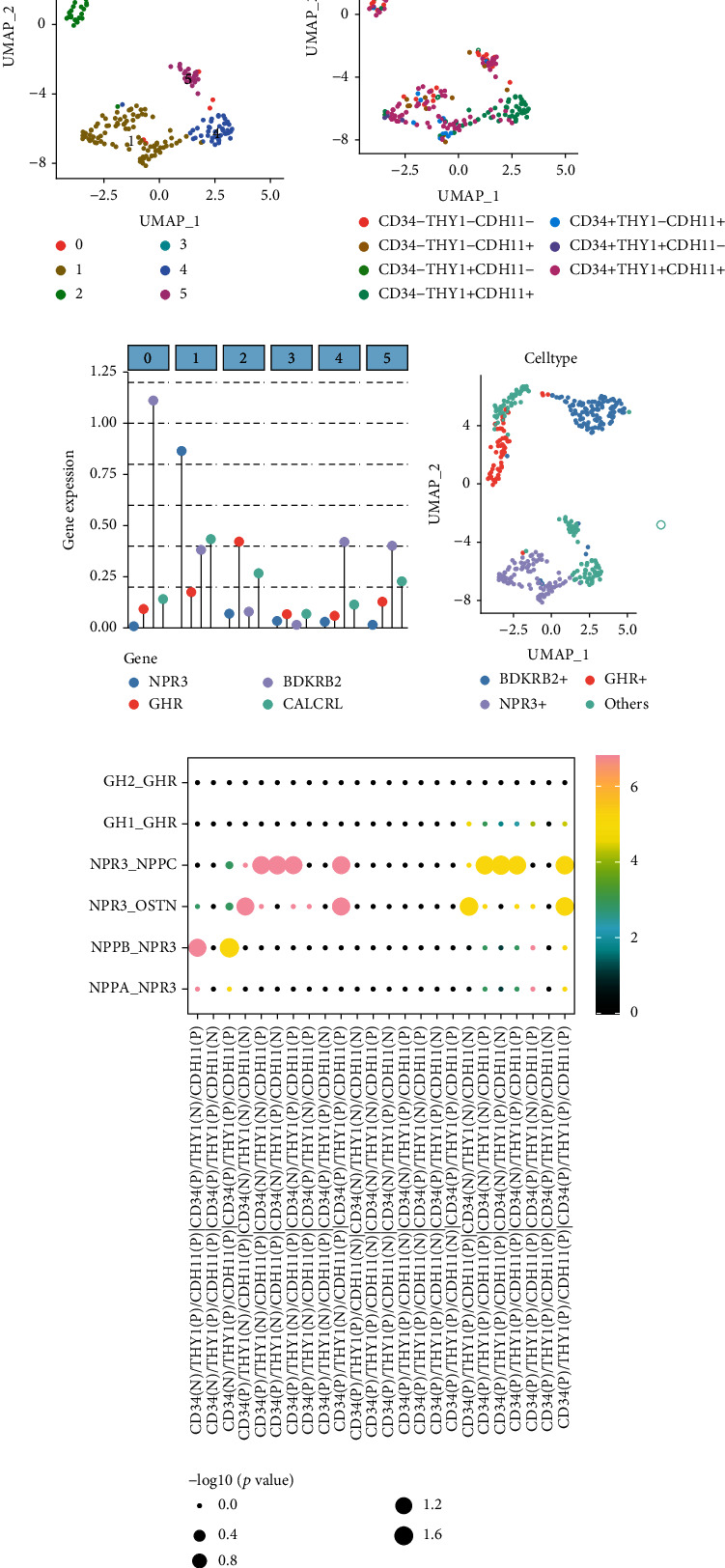
Clustering results and ligand–receptor interactions in GSE109449. (a) UMAP results for six clusters after dimensional reduction clustering. (b) Cellular annotation of UMAP based on marker genes from previous articles. (c) Relative expression levels of neuropeptide mRNAs with significant expression differences. (d) Cell clusters were annotated with UMAP based on *BDKRB2*, *NPR3*, and *GHR* expression levels in (c). (e) Correlation heat map of receptor–ligand interactions. For example, NPPB released from CD34-THY1+CDH11+ cells interacted with the NPR3 receptor on CD34+THY1-CDH11+ and CD34+THY1+CDH11+ cells. GH1 released from CD34+THY1+CDH11+ cells interacts with the *GHR* on other cells. Abbreviations: UMAP: Uniform Manifold Approximation and Projection.

The UMAP plot of SFs revealed six cell clusters (Figure 2(a)). Three major cell surface proteins, namely, CD34, THY1, and CDH11, were used to annotate cell types in UMAP, and seven cell types were identified (Figure 2(b)). Furthermore, the relative expression of highly variable neuropeptide-related genes, namely, *NPR3*, *GHR*, *BDKRB2*, and *CALCRL*, was compared between cell clusters (Figure 2(c)). The expression of *NPR3* was significantly higher in cluster 2 than in other clusters. In addition, the expression of *GHR* was significantly higher in cluster 2, and the expression of *BDKRB2* was significantly higher in cluster 0. We found that the expression of *BDKRB2*, *NPR3*, and *GHR* was more pronounced compared to *CALCRL*. Therefore, cell annotations of *BDKRB2*, *NPR3*, and *GHR* were also demonstrated in the UMAP plot (Figure 2(d)).

### 3.3. Analysis of Neuropeptide-Related Ligands and Receptors

In these seven cell types, significantly different neuropeptides and their receptors were used to infer intercellular communication. As shown in Figure 2(e), the ligand–receptor interaction analysis reveals the pairing results of multiple neuropeptides and their receptors. *NPR3*, *NPPC*, *OSTN*, *NPPB*, and *NPPA* were found to interact in the communication cascade of SFs. In addition, there may be an interaction between CD34+THY1+CDH11+ cells and *GHR* from other cells.

### 3.4. Machine Learning Predicts Neuropeptide-Related m6A Factors

**Figure 3:**
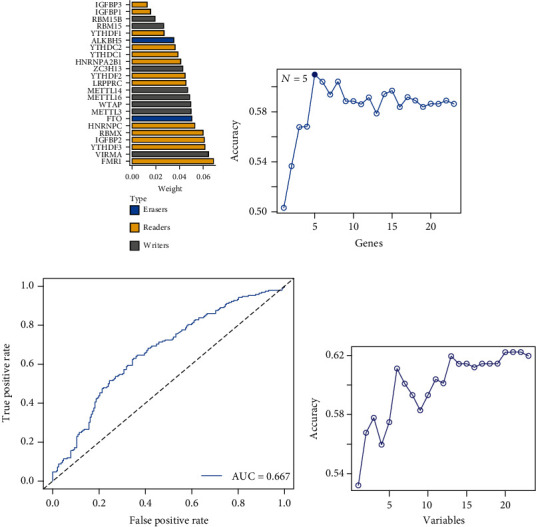
A machine learning-based gene screening process. (a) The random forest model of *GHR* prediction shows the importance of *GHR*-related 23 m6A enzymes. The genes are sorted by importance, and the m6A types to which they belong are shown in the legend. (b) SVM-RFE-based GHR prediction model for gene screening process. The final model accuracy was highest when five m6A regulatory genes were screened out. (c) ROC curves of SVM models constructed based on the screened five genes. (d) The variable selection process of the *NPR3* prediction model based on SVM-RFE showed the highest model prediction accuracy when 21 genes were included. Abbreviations: ROC: receiver operating characteristics; SVM-RFE: support vector machine-recursive feature elimination.

Using the random forest model, the ranking of the important m6A factors associated with *GHR* is shown in Figure 3(a). *FMR1*, *YTHDF3*, *IGFBP2*, and *RBMX* (readers) and *VIRMA* (writers) were identified as the top five significant m6A regulators. The results of the variable screening of the SVM-RFE model showed the highest accuracy of the model for predicting *GHR* when five m6A regulatory genes were screened out (Figure 3(b)), with an AUC of 0.667 (Figure 3(c)). Similarly, the *NPR3* expression prediction model was most accurate when based on 21 variables (Figure 3(d)).

### 3.5. *IGFBP2* and *METTL3* Are Identified as Important m6A Regulators

**Figure 4:**
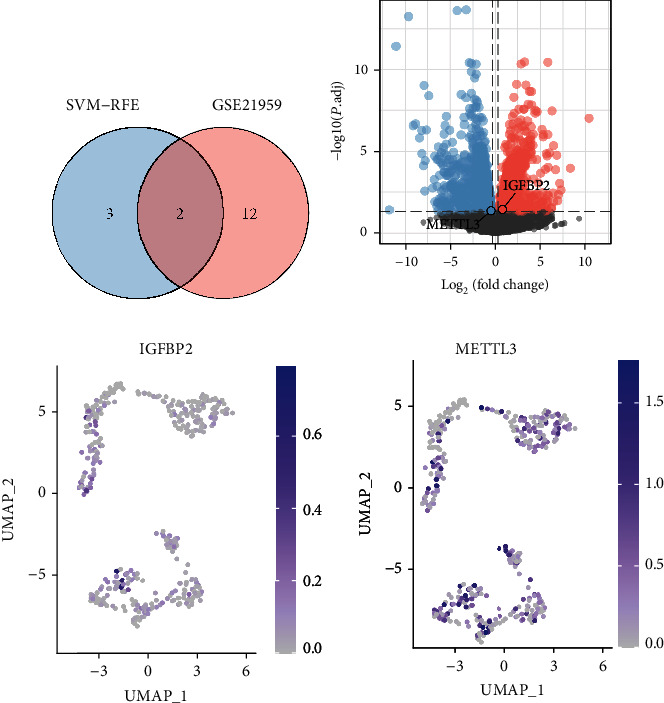
Combined analysis of screened m6A regulatory genes and DE mRNAs. (a) Venn plot showing the results of intersection analysis of SVM-RFE and DE mRNAs. A total of two m6A regulatory-related genes were screened. (b) Volcano plot showing the results of the differential analysis in bulk RNA. The two m6A regulatory genes are labelled in the graph. (c) Expression levels of *IGFBP2* and *METTL3* in UMAP. Abbreviations: DE: differentially expressed; SVM-RFE: support vector machine-recursive feature elimination.

**Figure 5:**
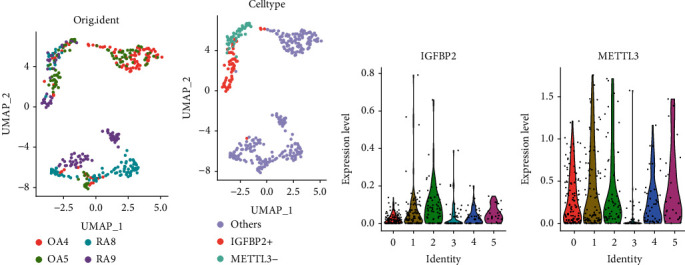
Expression characteristics of *IGFBP2* and *METTL3*. (a) UMAP of the initial sample information. (b) Upregulated *IGFBP2* and downregulated UMAP of *METTL3*. (c) The expression levels of *IGFBP2* in the six clusters. Among them, a slight increase in *IGFBP2* expression level was seen in cluster 2. (d) Expression levels of *METTL3* in six clusters. Among them, a decrease in *METTL3* expression level was seen in cluster 3.

The genes screened in the SVM-REF model were intersected with the DEGs in RA, and a total of two m6A regulatory genes were identified, namely, *METTL3* and *IGFBP2* (Figures 4(a) and 4(b)). The horizontal axis represents the fold change, with points further from the centre indicating a greater fold of difference; the vertical axis represents the FDR-adjusted *P* value, with points closer to the top indicating a more significant difference in expression between the two samples. However, in Figure 4(b), the difference between these two genes does not seem to be significant. But the next study revealed their potential significance. *METTL3* is an m6A writer, and *IGFBP2* is an m6A reader. The expression of *METTL3* and *IGFBP2* was assessed at the single-cell level using the UMAP algorithm (Figure 4(c)). *IGFBP2*+ fibroblasts were mainly found in RA9 and OA5 samples (Figure 5(a)). Volcanic map analysis revealed elevated expression of *IGFBP2* in cluster 2 (thought to be *IGFBP2*+) and downregulation of *METTL3* in cluster 3 (speculated to be *METTL3*-) (Figure 5(b)). Violin plots revealed the expression levels of *METTL3* and *GFBP2* in six cell clusters (Figures 5(c) and 5(D)). *METTL3* cells were present in both RA and OA samples. The combined analysis demonstrated in Figure 2(d) and Figure 5(b) indicated that both *IGFBP2* and GH2 were upregulated in cluster 2. Figures 2(c) and 5(d) demonstrate that the expression of *NPR3, GH2, BDKRB2* and *CALCRL* was significantly decreased in cluster 3 along with *METTL3* downregulation, suggesting that *METTL3* is an important regulator of the aforementioned four neuropeptide-related genes. Furthermore, in cluster 1, *NPR3* was significantly upregulated; however, no significant changes were observed in the expression of *IGFBP2* and *METTL3*, suggesting that other m6A factors may regulate posttranscriptional modifications of *NPR3*.

### 3.6. GSEA and Clinical Analysis of *IGFBP2* and *METTL3*

**Figure 6:**
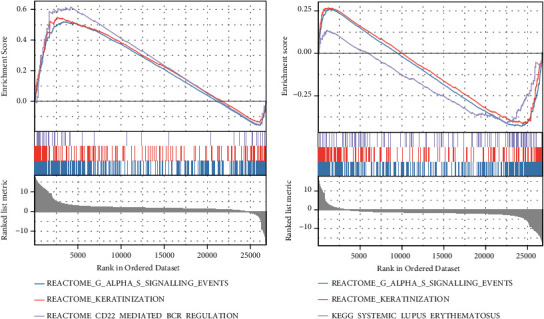
Gene set enrichment analysis of *IGFBP2* and *METTL3*. (a) Enrichment of upregulated functional pathways in samples with *IGFBP2* upregulation. (b) Downregulated functional pathways in samples with *METTL3* downregulation.

**Figure 7:**
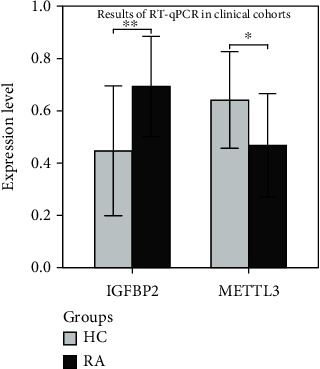
Results of the qRT-PCR in clinical cohorts: expression levels of IGFBP2 and METTL3 in healthy controls and rheumatoid arthritis synovial fibroblast samples. IGFBP2 was elevated in RA compared with HCs (*P* < 0.05). METTL3 was downregulated in RA compared with HCs (*P* < 0.05). Abbreviations: HC: health control; RA: rheumatoid arthritis; qRT-PCR: quantitative reverse transcription polymerase chain reaction.

GSEA was performed to further assess the possible functions of *IGFBP2* and *METTTL3* (Figure 6). *IGFBP1* upregulation was found to be accompanied by the upregulation of keratinisation and CD22-mediated BCR regulatory pathways. This finding suggests that *IGFBP2* is involved in keratinisation and immune behaviour in RA. G alpha(s) signalling, keratinisation, and systemic lupus erythematosus pathways were downregulated in cells with downregulated *METTL3*. This finding suggests that different m6A factors in RA play different or even opposite roles in different SFs. The qRT-PCR results of the clinical cohort revealed that *IGFBP2* was elevated in RA SFs compared with the healthy controls (HCs) (*P* = 0.008), and *METTL3* was downregulated in RA SFs compared with HCs (*P* = 0.027) (Figure 7). Therefore, we speculate that the m6A methylation-related genes *IGFBP2* and *METTTL3* may influence the interaction between SFs by regulating the neuropeptides *GHR* and *NPR2*, thus contributing to disease progression in RA.

## 4. Discussion

In this study, using single-cell analysis and machine learning, we determined the differential expression of *GHR* and *NPR2* in SFs. The m6A methylation-related genes *IGFBP2* and *METTTL3* are thought to influence the interaction between SFs by regulating the neuropeptides *GHR* and *NPR2*.

We determined the intercellular heterogeneity of SFs via single-cell RNA-seq analysis. Cell–cell communication simulations were performed to find possible neuropeptide-associated ligand–receptor pairs among the annotated SF subpopulations. The results revealed that the neuropeptide-related genes *GHR* and *NPR3* belonged to both cluster marker genes and screened ligand–receptor pairs. Subsequently, we used machine learning to predict m6A factors that may act on *GHR* and *NPR3*. In addition, RA-related genes were identified via differential analysis of RNA. Therefore, after the intersection analysis, we confirmed the possible important roles of *IGFBP2* and *METTL3*. *IGFBP2*+ fibroblasts were mainly found in RA and OA samples, which suggested heterogeneity in *IGFBP2* expression at the individual level. In cluster 3, *NPR3*, *GH2*, *BDKRB2*, and *CALCRL* were significantly decreased along with *METTL3* downregulation, suggesting that *METTL3* is an important regulator of the aforementioned four neuropeptide-related genes. In cluster 2, both *IGFBP2* and *GH2* were upregulated. In addition, in cluster 1, *NPR3* was significantly upregulated; however, no significant changes were observed in the expression of *IGFBP2* and *METTL3*, suggesting that other m6A factors regulate posttranscriptional modifications of *NPR3*. To the best of our knowledge, we report for the first time the possible role of *GH2* and *NPR3* in the progression of RA. Therefore, the significance and mechanism of action of the differential expression of *GH2* and *NPR3* between different SFs require further analysis and validation.

METTL3 is an important m6A catalase. Previous studies have shown that *METTL3* can enhance RNA stability, thereby affecting cell division [39]. *METTL3* reduction can also result in m6A dysregulation leading to neurodegeneration in Alzheimer's disease [40]. In our study, we found that *METTL3* downregulation was accompanied by a significant decrease in neuropeptide-related mRNAs, such as *NPR3*, *GH2*, *BDKRB2*, and *CALCRL* in cluster 3.

*IGFBP2* was found to be elevated in serum samples from the RA cohort, suggesting that *IGFBP2* may have an important role in the pathogenesis of RA [41]. Our study further demonstrates that *IGFBP2* is also significantly heterogeneous across individuals and subgroups of SFs. Moreover, we observed that *IGFBP2* and *GH2* were upregulated in cluster 2, suggesting that *IGFBP2* acts through the upregulation of *GH2*.

The development of single-cell sequencing has offered new methods to understand and study the diversity and function of cell subpopulations [21]. In this study, we examined the potential mechanisms of neuropeptides and m6A in RA using single-cell analysis and machine learning. However, data were not validated at the single-cell level. And the cell–cell communication analysis was performed on SFs, which may not be completely consistent with the way neuropeptides are regulated in vivo. In this study, we proposed a new approach to investigating the pathogenesis of RA. In the future, combined with spatial transcriptomic analysis, single-cell technology will help to better understand this disease. Better experimental designs and clinical studies to understand and confirm the involvement of these factors in RA development were still required.

## 5. Conclusion

We discovered neuropeptide-related molecules and m6A factors with key regulatory activities in RA with the help of single-cell analysis and machine learning. *GHR* and *NPR2* may play a role in the pathophysiological functional transformation of SFs. Through machine learning, the m6A factors, *METTL3* and *IGFBP2*, were found to regulate *GHR* and *NPR2* potentially. In clinical cohorts, *IGFBP2* was elevated in RA SFs, while *METTL3* was downregulated. This study may provide a basis for future studies to identify pathogenic cell subpopulations in RA and other complex diseases.

## Data Availability

The public datasets generated and analysed during the present study are available from GSE109449 and GSE21959.

## Abbreviations

UMAP: Uniform Manifold Approximation and Projection for Dimension Reduction
RA: Rheumatoid arthritis
m^6^A: N6-Methyladenosine
SVM-RFE: Support vector machine-recursive feature elimination
SF: Synovial fibroblasts
FLS: Fibroblast-like synoviocytes.
